# Mineral, trace element, and toxic metal concentration in hair from dogs with idiopathic epilepsy compared to healthy controls

**DOI:** 10.1111/jvim.16698

**Published:** 2023-04-06

**Authors:** Sarah Rosendahl, Johanna Anturaniemi, Tiina‐Kaisa Kukko‐Lukjanov, Kristiina A. Vuori, Robin Moore, Manal Hemida, Anne Muhle, Anna Hielm‐Björkman

**Affiliations:** ^1^ Department of Equine and Small Animal Medicine, Faculty of Veterinary Medicine University of Helsinki Helsinki Finland; ^2^ Department of Veterinary Biosciences, Faculty of Veterinary Medicine University of Helsinki Helsinki Finland; ^3^ Evidensia Espoo Animal Hospital Espoo Finland

**Keywords:** arsenic, canine, copper, diet, ICP‐MS, oxidative stress, phosphorus, potassium bromide, selenium, zinc

## Abstract

**Background:**

Altered trace element status is associated with epilepsy in humans and dogs with idiopathic epilepsy (IE).

**Objectives:**

Compare hair element concentrations in epileptic and healthy dogs.

**Animals:**

Sixty‐three dogs with IE (53 treated, 10 untreated) and 42 controls.

**Methods:**

Case‐control study using ICP‐MS to determine hair calcium, magnesium, phosphorus, sodium, potassium, iron, copper, manganese, zinc, selenium, chromium, lead, mercury, cadmium, arsenic, aluminum, and nickel concentration. Groups were compared using nonparametric tests. Results were controlled for diet, sex, age, and hair color using generalized linear mixed models.

**Results:**

Compared to healthy controls, dogs with IE had lower hair phosphorus (mean ± SD; IE: 286.19 ± 69.62 μg/g, healthy: 324.52 ± 58.69 μg/g; *P* = .001), higher hair copper (IE: 10.97 ± 3.51 μg/g, healthy: 8.41 ± 1.27 μg/g; *P* < .001), zinc (IE: 158.25 ± 19.64 μg/g, healthy: 144.76 ± 32.18 μg/g; *P* < .001), copper/zinc ratio (IE: 0.07 ± 0.02, healthy: 0.06 ± 0.01; *P* = .003), selenium (IE: 1.65 ± 0.43 μg/g, healthy: 0.94 ± 0.73 μg/g; *P* < .001), and arsenic (IE: 0.40 ± 0.78 μg/g, healthy: 0.05 ± 0.08 μg/g; *P* < .001). When comparing treated and untreated epileptic dogs with healthy dogs, the differences in phosphorus and selenium remained significant for both groups, whereas the differences in copper, zinc, and arsenic were significant only for treated dogs. Potassium bromide treatment was strongly associated with high hair arsenic (*P* = .000).

**Conclusions and Clinical Importance:**

Altered trace element status could be involved in the pathophysiology of IE in dogs. Antiseizure drugs might affect trace element and arsenic metabolism.

**Abbreviations**
ASD: antiseizure drug
Al: aluminum
As: arsenic
Ca: calcium
Cd: cadmium
Cr: chromium
Cu: copper
EEAH: Evidensia Espoo Animal Hospital
Fe: iron
GLMM: generalized linear mixed model
Hg: mercury
HUAH: Helsinki University Animal Hospital
ICP‐MS: inductively coupled plasma mass spectrometry
IE: idiopathic epilepsy
K: potassium
KBr: potassium bromide
LOD: limit of detection
Mg: magnesium
Mn: manganese
Na: sodium
Ni: nickel
P: phosphorus
Pb: lead
Se: selenium
Zn: zinc

## 1 INTRODUCTION

Epilepsy is considered the most common neurological disorder in dogs with an estimated prevalence of 0.6%‐0.75% in the general dog population.^1^, ^2^ After excluding reactive seizures caused by metabolic disorders or intoxication and structural epilepsy because of intracranial lesions, most epileptic dogs are diagnosed with idiopathic epilepsy (IE), where the underlying cause of seizures either remains unknown or is attributed to a confirmed or suspected genetic origin.^3^, ^4^, ^5^ Today, IE is viewed as a complex and multifactorial disease with multiple genes and environmental triggers.^3^ One‐third of cases remain unresponsive to antiseizure drug (ASD) treatment,^6^ and many have behavioral changes,^7^ resulting in lowered quality of life. Moreover, a shortened life expectancy is often reported for dogs with IE.^8^

Trace elements are essential for brain health by participating in enzymatic activities, mitochondrial functions, myelination, synaptogenesis and plasticity, and neurotransmission. Their deficiency or excess can result in neurodegeneration, inflammation, and oxidative stress, which contribute to neurological disorders and behavioral changes.^9^ An extensive body of research indicates that humans with epilepsy have altered trace element status, most commonly lower selenium (Se) and zinc (Zn), compared to controls,^10^, ^11^, ^12^, ^13^, ^14^, ^15^, ^16^ and that rebalancing using supplements can lead to a reduction in seizure frequency.^17^ There is evidence of trace element alterations in dogs in that dogs with IE have higher serum copper (Cu), Zn, Se, and manganese (Mn) concentrations, suggesting a role for these elements in the pathophysiology of epilepsy.

To determine trace element status in epileptic people or dogs, most studies have used serum or blood analysis. However, blood‐based samples are prone to rapid fluctuation and homeostatic regulation, and therefore, hair analysis, which provides a reading of elements deposited in the cells and interstitial spaces of the hair over a 2‐3‐month period, has been considered a more stable method for estimating long‐term trace element status.^18^ For instance, serum levels reflect a transient value and analysis of hair or other tissues might give a more accurate reflection of the body's true stores. In addition, hair analysis has been proposed as a useful diagnostic tool for the early diagnosis of chronic diseases in humans and dogs.^19^, ^20^

Therefore, the objective of our study was to expand the current knowledge about trace element status in dogs with IE by measuring their concentrations in hair. We hypothesized that epileptic dogs have altered trace element status that might be involved in the pathogenesis of IE and that certain elements might be affected by ASDs.

## 2 MATERIALS AND METHODS

### 2.1 Animals and study design

This was a case‐control study that included epileptic and healthy companion dogs living in their home environment in Finland. Dogs with a diagnosis of IE were recruited to give hair samples. Part of the dogs were regular clients of board‐certified neurologists at 2 small animal veterinary hospitals: Evidensia Espoo Animal Hospital (EEAH) and Helsinki University Animal Hospital (HUAH). To increase sample size, dog owners were also asked, via HUAH's Facebook page and other local dog groups, to send hair samples by post from dogs that have been diagnosed with IE. If they also had healthy dogs living in the same household, those dogs were recruited as controls. The minimum criteria for IE diagnosis in our study were a history of recurrent epileptic seizures (minimum 2), the first seizure occurring between 6 months‐6 years of age, and no detectable abnormalities on interictal clinical and neurological examination, complete blood cell count, and serum biochemistry profile. In addition to these minimum criteria, part of the dogs’ diagnostic workups also included additional tests, which are presented in Table 1. Dogs that were older than 3 years of age with no current or previous signs of neurological disease and no detected abnormalities in the clinical examination, complete blood count, or serum biochemistry profile served as controls. The majority of control dogs were selected from our previous data on hair and blood elements in healthy dogs.^21^ The extra control dogs that were recruited to send hair samples by post were healthy according to owner questionnaire data, but did not undergo clinical examination or blood testing. The exclusion criteria for both groups were pregnancy or lactation. All dog owners filled out an online questionnaire about basic information, health status, and feeding of the dog, and in the case that it was an epileptic dog, detailed questions about epilepsy. The dogs were grouped into raw, dry, and mixed diet groups based on their current diet: raw if their diet consisted of 80% or more of raw food, dry if their diet consisted of 80% or more of dry food, and mixed if their diet consisted of a mix of dry, raw, home‐cooked and/or canned food. In this study, raw diets were defined as various types of unprocessed animal meat and by‐products, and dry diets were defined as various commercially provided processed (often extruded) kibbles. Home‐cooked food was defined as foods cooked at home, including human food leftovers and/or snacks. Nutrient composition or ingredient lists were not collected for any of the diets. The study protocol received ethical approval by the Animal Experiment Board in Finland (ELLA; permit number: ESAVI/452/2020). All dog owners gave written informed consent for their dogs to participate in the study. Human ethics approval was not needed for this study.

**TABLE 1:** Tests included in the diagnostic workup of the epileptic dogs (N = 63).

Test	Number of dogs that had the test (n)
Complete blood cell count^a^ ^,^ ^b^	63
Basic serum biochemistry^b^ ^,^ ^c^	63
MRI	22
Additional blood testing^d^	16
Bile acid stimulation test	15
Vector‐borne pathogens^e^	7
Urinalysis	6
CSF analysis	3
EEG	3
Blood ammonia	2
Full thyroid panel^f^	1

Abbreviations: CSF, cerebrospinal fluid; EEG, electroencephalogram; MRI, magnetic resonance imaging.

^a^ Leucocytes, erythrocytes, hemoglobin, hematocrit, mean cell volume, mean cell hemoglobin, mean corpuscular hemoglobin concentration, and thrombocytes.

^b^ Included in the minimum criteria for IE diagnosis.

^c^ Alkaline phosphatase, alanine aminotransferase, albumin, total bilirubin, phosphate, glucose, potassium, sodium, calcium, cholesterol, creatinine, protein, and urea.

^d^ Reticulocytes, total thyroxine, symmetric dimethylarginine, chloride, gamma glutamyl transferase, aspartate aminotransferase, glutamate dehydrogenase, globulin, a‐amylase, lipase, fructosamine, muscle creatine kinase, magnesium, triglycerides, c‐reactive protein, basophils, eosinophils, segmented neutrophils, lymphocytes, and monocytes.

^e^ Anaplasma, Lyme disease, Ehrlichia, and heartworm.

^f^ Thyroxine, free thyroxine, thyrotropin, and thyroxine/thyrotropin.

### 2.2 Hair samples

A portion of the hair samples was collected by the main author or another veterinarian in a small animal clinic setting (n = 54) and another part was collected by the dog owners in their home environment (n = 53). In the latter case, dog owners were provided with disposable A12t Dilutus 80% ethanol disinfectant wet wipes (Berner Pro, Helsinki, Finland), paper envelopes, and detailed written and video instructions on how to take the sample. All samples were taken from the neck area. The hair was cleaned with A12t Dilutus 80% ethanol disinfectant (Berner Pro, Helsinki, Finland) and cut close to the skin using clean stainless‐steel scissors. The distal part of the sample was discarded, leaving only about the 3 cm of hair that had been closest to the skin. If the dog's hair was shorter than 3 cm, the full length of the hair was used. The total amount of hair in 1 sample was approximately 125 mg. Based on visual classification, the samples were classified as having either dark (70%‐100% of the sample was black or brown) or light (70%‐100% of the sample was white, cream/beige, red, or gray) color. This did not however always correspond to the dog's overall coat color. Hair samples were put in individual paper envelopes and, if the sample was taken by a dog owner, it was sent by post to the main author, whereafter all samples were stored at room temperature until analysis. The samples were sent to Accutrace Laboratories, Inc. (Phoenix, Arizona) for analysis of calcium (Ca), magnesium (Mg), phosphorus (P), sodium (Na), potassium (K), iron (Fe), Cu, Mn, Zn, Se, chromium (Cr), lead (Pb), mercury (Hg), cadmium (Cd), arsenic (As), aluminum (Al), and nickel (Ni). At the laboratory, the samples were placed in plastic cups and cut into small pieces using titanium scissors. Samples were then weighed, and 40 mg of unwashed hair was digested for 10 hours in PTFE reflux tubes containing a combination of nitric and perchloric acid (TraceMetal Grade, Fisher Chemical, Waltham, Massachusetts) in an open vessel and using a hot block/plate (Thermo Scientific model 2200) set at 188°C. After digestion, each sample was reconstituted to 2 mL using laboratory grade deionized water, and then analyzed with inductively coupled plasma mass spectrometry (ICP‐MS, Perkin Elmer nexION 2000B). A daily maintenance run was performed on the ICP‐MS instrument before analyzing the samples (Supplementary Material 1). To ensure accuracy of the results, both internal and external quality control (QC) procedures were implemented. Internal QC included calibration of the system twice per sample run using standard solutions. Homium and Thulium were used as single element internal standards with recovery (%) generally running between 95 and 105 for controls and case samples. The quality of the calibration was checked by running blanks and in‐house hair standards before running samples, after running all samples, and periodically every 20 samples. External QC was assured by the laboratory participating in 2 different external proficiency testing programs (Quebec Multielement External Quality Assessment Scheme (QMEQAS) and the Association of American Feed Control Officials (AAFCO)). Participation in both programs allowed external quality assessment for all elements included in this study. The laboratory has also been licensed by the Clinical Laboratory Improvement Amendments (CLIA #03D0641886) and has therefore met or exceeded the requirements of biennial inspections conducted by the Department of Health & Human Services (Washington, D.C.). The limit of detection (LOD) for all tested elements was 0.001 ppm or μg/g. The element concentrations were reported as μg/g. The Cu/Zn ratio was calculated, as it has been considered relevant in previous literature.^22^, ^23^

### 2.3 Statistical analysis

All statistical analyses were done using SPSS for Windows (version 27; IBM SPSS Statistics). Data normality was assessed using a Shapiro‐Wilk test. The characteristics of the study and control groups were compared using an independent sample *t*‐test for age (years) and weight (kg), and a chi‐square test for sex (male/female), hair color (light/dark), diet (dry/raw/mixed), living environment (rural/urban), and drinking water (tap/well water). First, a preliminary screen was done to evaluate which elements were significantly associated with diagnosis by using a Mann‐Whitney *U* test or a Kruskal‐Wallis test followed by pairwise comparisons with the Dunn‐Bonferroni approach. Elements with significant (*P* < .05) differences between epileptic and healthy dogs were then chosen for further analyses. Data were not normally distributed, and some sample subgroups were small. A generalized linear mixed model (GLMM) was therefore used to determine the associations of selected hair element concentrations as target variables with diagnosis, diet, sex, age, and/or hair color as fixed effects. The selection of tested variables was based on our previous study.^21^ Normal distribution with log link, a confidence level of 95%, robust estimation (robust covariances) and the Satterthwaite method for degrees of freedom calculation were used. Residual distributions were evaluated from histogram and Q‐Q plots. Statistical significance was set at *P* < .05 in all analyses.

## 3 RESULTS

### 3.1 Animals

A total of 78 epileptic dogs were recruited for this study. Of these, 15 were excluded: 6 because of late seizure onset, 4 because of having had only 1 seizure, 2 because of having an unclear epilepsy diagnosis, 1 because of having hypothyroidism, 1 because of having Chiari malformation and syringomyelia, and 1 because of abnormalities in the complete blood cell count and serum biochemistry profiles. We included 1 dog with seizure onset at 6.6 years of age as this dog's diagnostic workup included a brain MRI without any detected abnormalities. The control group included 31 dogs from our previous study on hair elements in healthy dogs^21^ and 11 dogs that were recruited from the same households as the epileptic dogs to send hair samples by post. Thus, the study included a total of 105 dogs: 63 dogs with IE and 42 controls. Of the epilepsy dogs, 28 were clients of board‐certified neurologists and 35 were recruited to send hair samples by post and were thus owner‐reported epilepsy cases and clients of unspecified veterinarians. In the latter case, the diagnosis was established by a specific and extensive disease related questionnaire (Supplementary Material 2) and by the fact that most of these dogs were receiving ASDs. In addition, we were able to watch seizure videos from 18 out of these 35 post recruited dogs. Tier confidence levels I or II was achieved in most epileptic dogs in our study, although we were not able to categorize all dogs because of the diversity of the diagnostic workups. The epileptic dogs were divided into subgroups of treated (n = 53) and untreated (n = 10). The most used ASDs were phenobarbital, potassium bromide (KBr), and imepitoin. Both epilepsy and control groups consisted of a large variety of breeds. Supplementary Material 3 presents, based on owner‐provided questionnaire data and/or retrospective data inspection from EEAH/HUAH data bases, detailed information about each dog's characteristics (breed, sex, age, weight) and diet, as well as for epileptic dogs, data on the diagnostic workup, ASD treatment, age of seizure onset, time since last seizure, and if the dog is showing 2 or more symptoms that are typical for focal or generalized seizures with tonic‐clonic movements and loss of or impaired consciousness (stiffness of limbs and neck, falling, lying down, twitching of muscles and limbs, turning the head in some direction, facial muscle twitching, chewing movements, urination, defecation, drooling, dilation of the pupils, rolling back of the eyes). To see if results were affected by genetic factors, we further stratified the epileptic dogs into 2 groups: 1 group with breeds considered epilepsy prone in literature,^3^, ^24^ having a reported family history of epilepsy, or a combination of these criteria (n = 42), and the other group with the remaining epileptic dogs (n = 21).

The characteristics of the study and control groups are presented in Table 2. The groups were similar regarding sex, weight, diet, hair color, living environment, and drinking water, whereas the mean age was slightly higher in the healthy dog cohort, since it included only dogs older than 3 years.

**TABLE 2:** Characteristics of the study and control groups.

Variable	Epileptic (n = 63)			Healthy (n = 42)	*P* values	
	All (n = 63)	Untreated (n = 10)	Treated (n = 53)		P1	P2
Age, years, mean (min‐max)^a^	5.34 (1.63‐11.60)	5.13 (2.42‐8.31)	5.38 (1.63‐11.60)	6.41 (3.00‐12.10)	.04	.11
Sex, n (%)^b^					.42	.56
Male	39 (61.9)	7 (70)	32 (60.4)	22 (52.4)		
Female	24 (38.1)	3 (30)	21 (39.6)	20 (47.6)		
Weight, kg, mean (min‐max)^a^	23.0 (1.0‐68.0)	24.3 (7.0‐57.0)	22.8 (1.0‐68.0)	22.90 (5.2‐50.1)	.96	.94
Diet, n (%)^b^					.1	.18
Dry^c^	32 (50.8)	6 (60)	26 (49.1)	20 (47.6)		
Raw^c^	8 (12.7)	2 (20)	6 (11.3)	12 (28.6)		
Mixed^d^	23 (36.5)	2 (20)	21 (39.6)	10 (23.8)		
Hair color, n (%)^b^					.16	.16
Light^e^	42 (66.7)	8 (80)	34 (64.2)	22 (52.4)		
Dark^f^	21 (33.3)	2 (20)	19 (35.8)	20 (47.6)		
Living environment, n (%)^b^					.32	.06
Rural	48 (76.2)	10 (100)	38 (71.7)	36 (85.7)		
Urban	15 (23.8)	0 (0)	15 (28.3)	6 (14.3)		
Drinking water, n (%)^b^					.07	.08
Tap water	55 (87.3)	8 (80.0)	47 (88.7)	30 (71.4)		
Well water	8 (12.7)	2 (20.0)	6 (11.3)	12 (28.6)		

*Note*: P1, all epileptic dogs vs healthy dogs. P2, untreated epileptic dogs vs treated epileptic dogs vs healthy dogs.

^a^ Independent samples *t*‐test/ANOVA.

^b^ Chi‐square test.

^c^ 80% or more of the total diet.

^d^ Mix of dry, raw, home‐cooked and/or canned food.

^e^ 70% to 100% of the hair sample was white, cream/beige, red, or gray.

^f^ 70% to 100% of the hair sample was black or brown.

### 3.2 Hair element concentrations between groups

**FIGURE 1:**
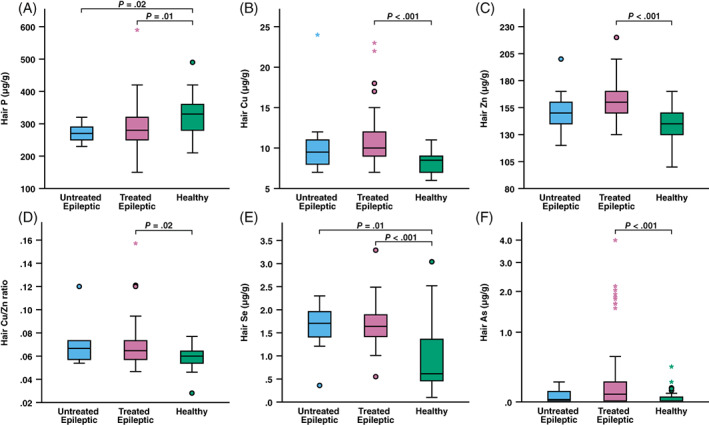
Box and whisker plot depicting hair concentrations of phosphorus (A), copper (B), zinc (C), copper/zinc ratio (D), selenium (E), and arsenic (F) in untreated epileptic (n = 10), treated epileptic (n = 53), and healthy (n = 42) dogs. Boxes represent the interquartile range (IQR) from the 25th to 75th percentile, while the horizontal lines in each box represent the median. Whiskers indicate the range of the data, excluding outliers, which are indicated by circles (o) when >1.5 times the IQR and by asterisks (*) when >3 times the IQR. To improve box plot scaling, 1 healthy dog with a zinc concentration of 320 μg/g is not depicted in (C), although it was included in analyses, and hair arsenic is shown in a logarithmic scale.

Mean hair element concentrations in the study and control groups are presented in Table 3. The table also presents mean hair element concentrations in healthy dogs from 2 previous studies. Epileptic dogs had significantly lower hair P compared to healthy dogs (*P* = .001), and this difference was significant also when looking at untreated (*P* = .02) and treated (*P* = .01) epileptic dogs separately (Figure 1A). The GLMM showed that sex and hair color also affected hair P, being higher in females (*P* = .01) and dark‐haired dogs (*P* < .001). However, after adjusting for these cofactors, the difference in hair P was also significant because of disease status (*P* = .04).

**TABLE 3:** Mean hair element concentrations (μg/g) in epileptic dogs (n = 63), divided into untreated (n = 10) and treated (n = 53), and healthy dogs (n = 42).

Hair element (μg/g)	Epileptic (n = 63) mean ± SD (min‐max)			Healthy (n = 42) mean ± SD (min‐max)	*P* values		
Reference values from the literature	All (n = 63)	Untreated (n = 10)	Treated (n = 53)		P1	P2	P3
Ca	614.76 ± 461.85 (140‐2170)	576.00 ± 546.71 (160‐1640)	622.08 ± 449.73 (140‐2170)	722.38 ± 682.42 (130‐3590)	.7	NS	NS
508.75 ± 383.52 (100‐1950)^a^							
588.00 ± 307.85^b^							
Mg	139.37 ± 99.58 (20‐450)	136.00 ± 105.75 (40‐340)	140.00 ± 99.42 (20‐450)	176 ± 140.01 (30‐590)	.23	NS	NS
155.60 ± 126.24 (30‐590)^a^							
124.00 ± 66.53^b^							
P	286.19 ± 69.62 (150‐590)	270.00 ± 29.06 (230‐320)	289.25 ± 74.65 (150‐590)	324.52 ± 58.69 (210‐490)	.001***	.02*	.01*
311.80 ± 61.90 (210‐490)^a^							
260.00 ± 37.71^b^							
Na	2794.29 ± 1844.42 (200‐8820)	3147.00 ± 2085.71 (980‐7530)	2727.74 ± 1809.62 (200‐8820)	2626.19 ± 1930.04 (470‐10 460)	.45	NS	NS
1793.88 ± 1127.90 (110‐5130)^a^							
2854.00 ± 859.25^b^							
K	175.24 ± 99.19 (30‐410)	176.00 ± 120.20 (60‐410)	175.09 ± 96.07 (30‐400)	215.48 ± 162.20 (40‐730)	.39	NS	NS
167.71 ± 110.50 (40‐560)^a^							
142.00 ± 76.85^b^							
Fe	39.44 ± 38.37 (11‐241)	63.20 ± 68.36 (11‐241)	34.96 ± 28.60 (13‐173)	35.86 ± 24.36 (13‐138)	.8	NS	NS
33.48 ± 17.50 (13‐98)^a^							
16.20 ± 4.94^b^							
Cu	10.97 ± 3.51 (7‐24)	10.90 ± 4.86 (7‐24)	10.98 ± 3.25 (7‐23)	8.41 ± 1.27 (6‐11)	< .001***	.1	< .001***
8.34 ± 1.35 (6‐13)^a^							
8.80 ± 1.03^b^							
Zn	158.25 ± 19.64 (120‐220)	151.00 ± 22.34 (120‐200)	159.62 ± 19.01 (130‐220)	144.76 ± 32.18 (100‐320)	<.001***	.74	<.001***
135.60 ± 15.54 (100‐170)^a^							
136.00 ± 8.43^b^							
Cu/Zn ratio	0.07 ± 0.02 (0.05‐0.16)	0.07 ± 0.02 (0.05‐0.12)	0.07 ± 0.02 (0.05‐0.16)	0.06 ± 0.01 (0.03‐0.08)	.003**	.15	.02*
Mn	0.96 ± 1.16 (0.20‐6.78)	1.83 ± 2.07 (0.22‐6.78)	0.79 ± 0.84 (0.20‐5.20)	1.33 ± 1.97 (0.17‐10.35)	.34	NS	NS
0.69 ± 0.44 (0.17‐2.31)^a^							
0.39 ± 0.17^b^							
Se	1.65 ± 0.43 (0.36‐3.29)	1.62 ± 0.56 (0.36‐2.30)	1.65 ± 0.41 (0.55‐3.29)	0.94 ± 0.73 (0.10‐3.04)	<.001***	.01*	<.001***
0.53 ± 0.24 (0.10–1.33)^a^							
1.01 ± 0.19^b^							
Cr	0.90 ± 0.49 (0.45‐3.08)	1.02 ± 0.34 (0.48‐1.51)	0.88 ± 0.51 (0.45‐3.08)	0.92 ± 0.50 (0.42‐3.53)	.44	NS	NS
0.88 ± 0.25 (0.42‐1.73)^a^							
1.07 ± 0.20^b^							
Pb	0.13 ± 0.19 (0.03‐1.50)	0.29 ± 0.44 (0.04‐1.50)	0.10 ± 0.06 (0.03‐0.31)	0.17 ± 0.23 (0.01‐1.37)	.5	NS	NS
0.09 ± 0.06 (0.01‐0.30)^a^							
0.06 ± 0.05^b^							
Hg	0.16 ± 0.22 (0.01‐1.41)	0.31 ± 0.45 (0.01‐1.41)	0.13 ± 0.13 (0.02‐0.78)	0.17 ± 0.32 (0.02‐1.89)	.3	NS	NS
0.08 ± 0.04 (0.01‐0.21)^a^							
0.08 ± 0.03^b^							
Cd	0.02 ± 0.03 (0.01‐0.2)	0.02 ± 0.02 (0.01‐0.07)	0.02 ± 0.03 (0.01‐0.2)	0.01 ± 0.01 (0.01‐0.04)	.48	NS	NS
0.01 ± 0 (0.01‐0.01)^a^							
0.02 ± 0.0^b^							
As	0.40 ± 0.78 (0.01‐3.99)	0.07 ± 0.08 (0.01‐0.22)	0.46 ± 0.83 (0.01‐3.99)	0.05 ± 0.08 (0.01‐0.42)	<.001***	1	<.001***
0.02 ± 0.02 (0.01–0.09)^a^							
0.09 ± 0.07^b^							
Al	27.47 ± 36.33 (4.60‐210.80)	45.71 ± 62.07 (4.70‐210.80)	24.02 ± 28.83 (4.60‐164.50)	22.54 ± 18.22 (6.70‐105.40)	.58	NS	NS
21.42 ± 12.93 (6.70‐63.30)^a^							
4.82 ± 3.27^b^							
Ni	0.65 ± 0.90 (0.08‐4.53)	1.23 ± 1.64 (0.10‐4.53)	0.54 ± 0.65 (0.08‐3.15)	0.52 ± 1.09 (0.06‐6.23)	.03*	.04*	.27
0.24 ± 0.17 (0.06‐0.90)^a^							
0.10 ± 0.07^b^							

*Note*: Asterisks indicate significant differences between groups: *P < .05, **P < .01, ***P < .001. NS: no significant difference between groups. P1: All epileptic dogs vs healthy dogs. P2: Untreated epileptic dogs vs healthy dogs. P3: Treated epileptic dogs vs healthy dogs.

^a^ Rosendahl et al,^21^ mean ± SD (min‐max).

^b^ So et al,^25^ mean ± SD.

Hair Cu was significantly higher in epileptic compared to healthy dogs (*P* < .001). Both untreated and treated epileptic dogs had higher hair Cu compared to healthy dogs, although the difference reached statistical significance only in the treated (*P* < .001), but not in the untreated (*P* = .1) dogs (Figure 1B). Likewise, hair Zn was significantly higher in epileptic compared to healthy dogs (*P* < .001), and the difference was significant in the treated (*P* < .001), but not in the untreated (*P* = .74) dogs (Figure 1C). In the GLMM's, disease status was the only significant factor for both Cu and Zn, after considering other cofactors. As we saw a moderate, close to strong, correlation (R = 0.642) between hair Cu and Zn, we also made more complex models where Zn was included as a covariate in the Cu model, and vice versa. In the Cu model, disease status was still significant (*P* < .001) after adjusting for hair Zn (*P* = .01). However, in the Zn model, disease status lost its significance after adjusting for Cu. We calculated the Cu/Zn ratio and found that it was significantly higher in epileptic compared to healthy dogs (*P* = .003), although only reaching statistical significance in the treated (*P* = .02), but not in the untreated (*P* = .15) dogs (Figure 1D).

Hair Se was significantly higher in epileptic compared to healthy dogs (*P* < .001), and in this case, there was a clear significant difference for both untreated (*P* = .01) and treated (*P* < .001) dogs (Figure 1E). In the GLMM, disease status was the only significant factor.

Epileptic dogs had significantly higher hair As compared to healthy dogs (*P* < .001), and when looking at subgroups, this difference was only significant in the treated dogs (*P* < .001; Figure 1F). Among these, 10 dogs were identified as extreme outliers, and they were all treated with KBr. The mean hair As concentration of all dogs treated with KBr (n = 13; 1.67 μg/g ± 0.95; 0.16‐3.99) was 24‐fold higher than that of dogs treated with other ASDs (n = 40; 0.07 μg/g ± 0.07; 0.01‐0.30) and 36‐fold higher than that of healthy dogs. When added as a cofactor in the GLMM, KBr treatment was strongly significant (*P* = .000), whereas disease status lost its significance (*P* = .142).

Hair Ni was significantly higher in epileptic compared to healthy dogs (*P* = .03), although when looking at subgroups the difference was significant only in the untreated (*P* = .04) but not in the treated (*P* = .27) dogs. However, in the GLMM, disease status did not have a significant effect on hair Ni even if it was added to the model alone.

For Ca, Mg, Na, K, Fe, Mn, Cr, Pb, Hg, Cd, and Al no significant differences were found between epileptic and healthy dogs. However, we observed that untreated epileptic dogs had lower means of the minerals Ca, Mg, and K, and higher mean Na, as well as higher means of the toxic metals Pb, Hg, and Al compared to the healthy dogs. However, there was a large variation between dogs (large SD of means) which might have affected these results not being statistically significant. Genetic factors did not have any significant effect on the results (data not shown).

## 4 DISCUSSION

The present study supports the role of altered trace element status in dogs with IE. Dogs with IE had lower hair P and higher hair Cu, Zn, Se, and As, as well as a higher Cu/Zn ratio, compared to healthy controls. Alterations in P and Se were significant for both treated and untreated epileptic dogs, suggesting a possible role for these elements in the pathophysiology of IE. Treatment with KBr was associated with a significantly elevated hair As.

Epileptic dogs, both treated and untreated, had significantly lower hair P compared to healthy dogs. Phosphorus plays an essential role in energy metabolism as a component of adenosine triphosphate (ATP).^26^ Energy deficit and reduced ATP production have been associated with mitochondrial dysfunction and epilepsy.^27^ To our knowledge, this is the first study to report altered hair P in epileptic dogs. As the hair P concentration is not considered to reflect dietary P intake,^28^, ^29^ the reason for our finding might be related to other, for example, metabolic, factors.

Hair Cu was significantly higher in epileptic compared to healthy dogs, although the difference only reached statistical significance in treated, but not in untreated epileptic dogs. This is in line with the study by Vitale et al,^30^ where higher serum Cu was found in treated, but not in untreated epileptic dogs. In their study, treatment with phenobarbital, but not other ASDs, was associated with higher Cu, and this was attributed to an increased hepatic synthesis of ceruloplasmin induced by phenobarbital.^31^ When comparing hair Cu concentrations between epileptic dogs treated with phenobarbital and those treated with other ASDs, we did not find any significant difference (data not shown). According to human studies, Cu in hair remains unaffected by ASD treatment,^32^ unlike in serum.^33^ Furthermore, the mean hair Cu of epileptic dogs in our study was higher than that of healthy dogs in our previous study^21^ and in the study by So et al.^25^ In our previous study on 50 healthy dogs, the range for hair Cu concentration was 6‐13 μg/g, and in the current study, 8 epileptic dogs had hair Cu concentrations above 13 μg/g, with the highest being 24 μg/g. Contrary to our findings, a meta‐analysis from 2015 showed that humans with epilepsy had significantly lower hair Cu compared to controls.^16^ However, Shrestha et al^34^ reported higher hair Cu in 101 epileptic patients compared to 101 controls, and several studies have also reported higher serum Cu in epileptic patients.^14^, ^35^, ^36^, ^37^ Copper is an essential trace element needed for neurotransmitter synthesis, myelination of nerves, and modulation of synaptic activity, although in excess amounts it can lead to increased oxidative stress. Thus, Cu homeostasis has been considered important in the prevention of several neurodegenerative diseases such as Wilson's, Menkes, and Alzheimer's disease.^38^ By inhibiting Mg‐ATPase and Na, K‐ATPase, even low doses of Cu can lead to disturbed Na K homeostasis and seizures in rats.^39^ The cause for higher hair Cu in epileptic dogs in our study remains unknown. However, long‐term high intake of dietary Cu was reflected in the hair Cu content of humans, whereas plasma content remained unchanged,^40^ and therefore it is possible that our results reflect an excess intake of Cu in epileptic dogs. Copper concentration in dogs’ livers has increased over time as a result of changing environmental exposures such as the use of Cu water piping as well as changes in the Cu supplementation recommendations for commercial dog food set by AAFCO.^41^

Hair Zn was significantly higher in treated epileptic dogs compared to healthy dogs. There is higher serum Zn in uncontrolled epileptic dogs compared to healthy or untreated dogs.^30^ In comparison to previous data on healthy dogs,^21^, ^25^ the epileptic dogs, but also the control dogs in the current study had higher mean hair Zn. A meta‐analysis of human studies found higher serum Zn in untreated epileptic patients compared to healthy controls,^16^ although several studies report lower serum Zn in epileptic patients.^12^, ^15^, ^37^ Zinc homeostasis is critical for brain physiology, with adequate levels having a neuroprotective effect. In excess, Zn can become neurotoxic.^42^ In the brain, Zn can act either as an anticonvulsant or a proconvulsant by modulating a variety of receptors in the excitatory glutamatergic and inhibitory γ‐aminobutyric acid (GABA)‐ergic systems.^43^ Furthermore, the highest levels of Zn in the brain are found in the hippocampus, which supports the role of Zn in epilepsy.^43^

The Cu/Zn ratio has been considered both clinically more important than the concentration of either trace element by itself and a marker of oxidative stress and inflammation,^44^, ^45^ since excess Cu causes increased oxidative stress and neuroinflammation, whereas Zn acts as an antioxidative and anti‐inflammatory nutrient.^46^ This ratio was significantly higher in treated epileptic dogs compared to healthy dogs. A higher Cu/Zn ratio has also been found in the serum of children with idiopathic seizures.^36^ Elevated Cu and decreased Zn have also been associated with behavioral disorders such as hyperactivity, attention deficit disorders, depression, autism and schizophrenia^46^ and it would be interesting to see if the altered Cu and Zn status plays a role in neurobehavioral comorbidities in dogs with IE.^7^ However, further research is needed to clarify the role of Cu and Zn in IE in dogs.

Hair Se was significantly higher in epileptic compared to healthy dogs, and this was true for both treated and untreated subgroups. Higher serum Se is detected in treated epileptic dogs.^30^ The mean hair Se concentration in epileptic dogs in our study was 1.76 times higher than that of healthy dogs, more than 3 times higher than that of healthy dogs in our previous study,^21^ and 1.6 times higher than that of healthy dogs in another study.^25^ Hair Se concentration in 50 healthy dogs is 0.10‐1.33 μg/g, and in the current study, 51 of the 63 epileptic dogs (81%) had values above that range. Dogs with IE have significantly higher Se concentration also in whole blood.^47^ Human epileptic patients have lower serum Se compared to healthy people, based on a meta‐analysis,^48^ and Se supplementation reduces seizures.^49^ Dogs are less likely to be deficient in Se than humans, as they usually eat balanced commercial diets which follow micronutrient recommendations by the European Pet Food Industry (FEDIAF).^50^ Dog foods often contain excess amounts of Se, and it is suggested that Se content in dog food should be continuously monitored.^51^, ^52^, ^53^ Excess dietary Se intake in horses is reflected in the hair.^54^ Thus, as most of the dogs in our study were eating commercial diets, it is possible that our results reflect an excess of Se in some of these diets. Taken together, the current research suggest that Se plays a role in epilepsy in both dogs and humans, although there seem to be a discrepancy between species.

Epileptic dogs treated with KBr had significantly higher hair As compared to those treated with other ASDs and to healthy dogs. They also had elevated hair As compared to data from previous studies, with the mean (1.67 μg/g) being as much as 84 times higher compared to the mean of healthy dogs in our previous study (0.02 μg/g).^21^ In that study, we reported the hair As range in healthy dogs to be 0.01‐0.09 μg/g, and the results in the current study are clearly outside that range. Similarly, the mean in the current study was 19 times higher compared to the 0.09 μg/g reported elsewhere.^25^ Arsenic has a high affinity for keratin and hair can thus function as a secondary excretion route.^55^

Our study had several limitations. The main limitation was the small number of dogs included in the untreated epileptic group, which might have interfered with identifying significant relationships from the data. A limitation regarding the dogs that were recruited by post is that we do not know if their diagnosis was made by a certified neurologist or a general practitioner, although they had all received a diagnosis of IE and 7 of them had MRI examination of the brain which is usually done by specialists. Furthermore, since the control dogs were recruited from 3 years of age, we cannot know for sure that they will not develop epilepsy later in life. Another limitation was that we did not specify epileptic syndromes in this study. For example, seizure strength affects body metabolism and might thus potentially also affect trace element metabolism. Finally, we did not assess the dietary intake of the studied elements in our study, which would have made it possible to clarify whether the observed alterations were related to dietary intake or other reasons, such as metabolic or other changes. Since this study does not demonstrate a causal relationship between trace elements and IE in dogs it cannot serve as the basis for altering current treatment approaches.

In conclusion, this study strengthens the evidence of altered trace element status in dogs with epilepsy. Hair Se was significantly elevated also in drug‐naïve epileptic dogs, suggesting a role for Se in the pathogenesis of epilepsy in dogs. However, it remains unclear whether the observed alterations represent a contributing factor to developing epilepsy or seizures or if they are a consequence of the disease. The study also suggests that KBr might alter As metabolism in dogs.

## CONFLICT OF INTEREST DECLARATION

Authors declare no conflict of interest.

## OFF‐LABEL ANTIMICROBIAL DECLARATION

Authors declare no off‐label use of antimicrobials.

## INSTITUTIONAL ANIMAL CARE AND USE COMMITTEE (IACUC) OR OTHER APPROVAL DECLARATION

Approved by the Animal Experiment Board in Finland (ELLA; permit number: ESAVI/452/2020).

## HUMAN ETHICS APPROVAL DECLARATION

Authors declare human ethics approval was not needed for this study.

## Supporting information


**Supplementary Material 1.** ICP daily maintenance and monitoring form.

**Supplementary Material 2.** Canine epilepsy and other health information questionnaire.

**Supplementary Material 3.** Detailed information about study and control dogs.
