# Supplementary material for: Mineral, trace element, and toxic metal concentration in hair from dogs with idiopathic epilepsy compared to healthy controls
Source: J Vet Intern Med. 2023 Apr 6;37(3):1100–10. doi: 10.1111/jvim.16698 (PMC10229330; doi:10.1111/jvim.16698)
Supplement: Supplementary file 1 — Supplementary Material 1. ICP daily maintenance and monitoring form. [file JVIM-37-1100-s001.pdf]

Supplementary Material 1 ICP Daily Maintenance and Monitoring Form

ICP Daily Maintenance and Monitoring Form

The Performance of the ICP is monitored and assessed by performing the daily performance analysis on the instrument. The results of the performance test and any maintenance performed are logged and recorded daily. The following parameters are recorded on a log so that any trends, sudden changes or need for maintenance can be easily observed or anticipated.

	**Parameter**	**Acceptable limits**
1.	Beryllium ion intensity	>4,500
2.	Beryllium ion RSD	<2.5%
3.	Indium ion intensity	>80,000
4.	Indium ion RSD	<2.5%
5.	Uranium ion intensity	>60,000
6.	Uranium ion RSD	<2.5 %
7.	Ce++	<0.03
8.	CeO	<0.025
9.	Background Counts	<3 counts
10.	Nebulizer Flow range	0.8-1.1 ml/min
11.	Deadtime	30-60 ns
12.	Torch Temp.	<65 deg. C
13.	RF power	>l150
14.	Chiller coolant	Check level of coolant. Record ok or add
15.	Chiller pressure	Record chiller temp and pressure.
16.	Vacuum torr	< 1.0 X10 to the minus 6
17.	Neb clean	yes or no
18.	New pump tubing	yes or no
19.	Clean torch	yes or no
20.	Clean cones installed	yes or no (H2O2 for peroxide).
21.	X-Y Torch Position	Record in mm
22.	Pump oil check	Check color and level; record ok or chg
23.	Due1 detector calibration performed	yes or no
24.	QID calibration performed	yes or no
25.	Mass Calibration Performed	yes or no
26.	Record Kr and As counts from blank sample	High As and Kr may indicate bad argon
27.	Record Dewar Argon Level	Percent Full
