# Supplementary material for: Mineral, trace element, and toxic metal concentration in hair from dogs with idiopathic epilepsy compared to healthy controls
Source: J Vet Intern Med. 2023 Apr 6;37(3):1100–10. doi: 10.1111/jvim.16698 (PMC10229330; doi:10.1111/jvim.16698)
Supplement: Supplementary file 2 — Supplementary Material 2. Canine epilepsy and other health information questionnaire. [file JVIM-37-1100-s002.pdf]

Canine epilepsy and other health information questionnaire

Basic

Nickname

Official name, if any

Registration number, if any

Breed (if not, put "other")

Date of birth (dd.mm.yyyy)

Gender --Valitse tästä-- (male/female/neutered male/spayed female)

Weight (kg)

Color

The dog's health

Answer the questions to the best of your ability and add information about unusual conditions under "Additional information about the dog's health status".

Does your dog have or has had the following diseases/symptoms?

- ☐ I don't believe that my dog is sick
- ☐ Atopy/allergy
- ☐ Skin infections (e.g., rash, hot spot)
- ☐ Chronic intestinal symptoms (abdominal hypersensitivity, IBD, recurrent diarrhea)
- ☐ Frequent ear infections
- ☐ Furunculosis (inflammation between the toes)
- ☐ Demodicosis
- ☐ Fat deposits (lipoma)
- ☐ Tartar
- ☐ False pregnancy
- ☐ Anal gland infection
- ☐ Hypothyroidism
- ☐ Cushing's disease
- ☐ Addison's disease

☐ Pancreatic insufficiency (EPI)

☐ Epilepsy **(WHEN YOU CHECK THIS BOX, ADDITIONAL QUESTIONS ABOUT YOUR DOG’S EPILEPSY SHOW UP AT THE END OF THE QUESTIONNAIRE)**

☐ Diabetes

☐ Benign growths/nodules

☐ Cancer

☐ Recurrent urinary tract infections

☐ Kidney disease

☐ Liver disease

☐ Heart disease

☐ Eye disease

☐ Autoimmune disease

☐ Urinary stone disease

☐ Osteochondrosis dissecans

☐ Spondylosis

☐ Osteoarthritis

☐ Dilation and torsion of the stomach

☐ Pyometra

☐ Other disease/diseases (continued below)

Some other disease / other diseases, which / which ones?

If cancer, what type?

If autoimmune disease, what?

List your dog's regular medications here

Are the diseases diagnosed by a veterinarian, or are they your own thoughts? For each dog's illness, write separately whether they were diagnosed by a veterinarian or whether the diagnosis is based on your own assessment of the dog's symptoms.

Has your dog experienced traumatic events or periods in his life (e.g., has spent time at a puppy mill/kennel, accident, loss of owner or serious illness of a family member, violence, house change)?

Yes No

If you answered 'Yes', you can tell us what kind if you want

How often is the dog vaccinated?

- Never (if adult, juvenile or puppy)
- Never after puppy vaccinations
- Less often than every three years (if adult)
- Every three years (if adult)
- Yearly (if adult)
- 2-3 times during the first year (if puppy and young)

Do you use tick treatment for your dog?

Yes No

If you answered 'Yes', tell us what brand and type (tablet/collar/spot on) and how often it is used. Also tell me if side effects have appeared from the tick treatment.

Additional information about the dog's health status (e.g., what kind of heart disease, what kind of urinary stones, etc.)

Rate your dog’s

	Greatly reduced	Moderately reduced	Slightly reduced	Normal	Slightly increased	Moderately increased	Strongly increased
Activity	◌	◌	◌	◌	◌	◌	◌
Appetite	◌	◌	◌	◌	◌	◌	◌

Vomiting

None	Rarely (1 time/week)	Moderately (2-3 times/week)	Often (>3 times/week)
◌	◌	◌	◌

Stool composition

- ◌ Type 1: discrete, hard, hard-to-pass lumps
- ◌ Type 2: uniform, firm, distinctly lumpy
- ◌ Type 3: uniform, solid, cracks on the surface
- ◌ Type 4: uniform, soft and smooth
- ◌ Type 5: soft, separate lumps
- ◌ Type 6: fluffy, mushy
- ◌ Type 7: completely liquid/watery diarrhea

Types 1 and 2 are signs of constipation. Types 3 and 4 are normal, 5 and 6 mean softer stools than normal or mild diarrhea. Type 7 is diarrhoea/dropsy. See picture: https://fi.wikipedia.org/wiki/Stool types

My dog's poop contains

	Yes	No
Mucus	◌	◌
Blood	◌	◌

The composition of my dog's poop varies

Yes	No
◯	◯

If you answered 'Yes', please tell us how

Frequency of bowel movements

- ◯ Severely reduced (severe constipation)
- ◯ Moderately reduced (every two or three days)
- ◯ Slightly reduced (0-1 times/day)
- ◯ Normal (1-2 times/day)
- ◯ Slightly increased (2-3 times/day)
- ◯ Moderately increased (4-5 times/day)
- ◯ Strongly increased (>5 times/day)

My dog's frequency of bowel movements vary

Yes	No
◯	◯

If you answered 'Yes', please tell us how

Weight

	Is not	Mild (<5%)	Moderate (5-10%)	Severe (>10%)
Losing weight	◯	◯	◯	◯
Gaining weight	◯	◯	◯	◯

The dog's living environment

The dog lives mainly

- In the countryside on a farm
- In the countryside
- In a built-up area
- In an urban area

The dog spends the most time

- Indoors
- Outside (e.g., in a shelter)

Owner/other family member’s smoking habits

- Does not smoke
- Smokes, but only outside
- Smokes indoors

Additional information about the dog's living environment (write here if the dog has just come to you/moved, e.g., from the city to the country, if you spend weekends or summers in the country, etc.)

The dog's lifestyle

The dog is mainly a

- ☐ Companion dog
- ☐ Hunting dog
- ☐ Shepherd dog
- ☐ Hobby dog (e.g., agility)
- ☐ Dam (in kennel)
- ☐ Working dog

Something else, what? ________

My dog is for me a (choose up to 3)

- ☐ Dear friend
- ☐ Dear family member
- ☐ Hobby or work friend
- ☐ Hobby or work tool
- ☐ Breeding animal
- ☐ Utility animal
- ☐ Domestic animal

Something else, what? ________

I use my dog in training for everyday life/hobbies/work (only choose one)

- ○ Just rewarding
- ○ Mainly rewards
- ○ Equal amounts of reward and punishment
- ○ Mainly punishment
- ○ Just punishment
- ○ I don't know/I can't say

Dog walking/exercise without hobbies

	less than an hour	1-2 hours	more than two hours
The dog walks/exercises every day on weekdays	○	○	○
The dog walks/exercises a few times a week (e.g., on weekends) a day	○	○	○

The dog’s walks happen mainly

- ☐ On a road with a lot of traffic
- ☐ On a road with little traffic
- ☐ On a forest road
- ☐ In the dog park
- ☐ In the own yard
- ☐ In nature (in the forest, in the field)

Something else, what? ________

We do some kind of hobby with the dog weekly (or almost) [--Valitse tästä--] (Yes/No)

What hobby? ________

More information about the dog's lifestyle

The dog's diet

The composition of your dog's CURRENT diet (check that all your choices add up to 100%, Thank you!)

	0%	10 %	20 %	30 %	40 %	50 %	60 %	70 %	80 %	90 %	100 %	What (brand/type)?
Dry food	○	○	○	○	○	○	○	○	○	○	○	
Raw food/uncooked	○	○	○	○	○	○	○	○	○	○	○	
Home cooked/table scraps	○	○	○	○	○	○	○	○	○	○	○	
Dog sausage and canned food	○	○	○	○	○	○	○	○	○	○	○	

The dog's CURRENT diet includes

	never	1-6 times a year	monthly	weekly	daily	What (species/brand/type)
Slaughter waste from hunting	○	○	○	○	○	
Organs/offal	○	○	○	○	○	
Fish	○	○	○	◉	○	
Raw meat bones (e.g., pipe bones, chicken wings, cartilage bones)	○	○	○	◉	○	
Boiled rice	○	○	○	○	○	
Vegetables	○	○	○	○	○	
Berries	○	○	○	○	○	
Egg (raw)	○	○	○	○	○	
Egg (cooked)	○	○	○	○	○	
Dairy products	○	○	○	○	○	

	never	1-6 times a year	monthly	weekly	daily	What (species/brand/type)
Leather bones (white or beige)	◯	◯	◯	◯	◯	
Treats	◯	◯	◯	◯	◯	

Describe your dog's typical day, i.e., what the dog typically eats per day, all foods (brand/species/type, quantity, etc.)

Supplements used AT THIS MOMENT (at least used weekly)

- ☐ Animal-derived oils (e.g., fish oil)
- ☐ Plant-based oils (e.g., rapeseed oil, wheat germ oil, Nutrolin skin & coat)
- ☐ Probiotics
- ☐ Seaweed/kelp
- ☐ Egg shells
- ☐ Calcium
- ☐ Magnesium
- ☐ Zinc
- ☐ Multivitamin
- ☐ Vitamin A
- ☐ Biotin/B vitamin
- ☐ vitamin C
- ☐ Vitamin D
- ☐ Vitamin E
- ☐ Glucosamine/chondroitin sulfate

Some other supplement, what?

Which of the following options do you think best describes your dog's diet?

- ◯ The dog eats a low-fat diet (e.g., diet foods, low-fat commercial foods, low-fat dairy products, low-fat meats, etc.)
- ◯ The dog eats a high-fat diet (e.g., fatty meats, added oil, lard, high-fat dairy products, etc.)
- ◯ Neither (I would say that the dog eats a normal diet)

Here you can clarify the answer to the previous question by telling which low-fat or high-fat products you give your dog?

How many times a day do you feed your dog?

- ◯ 1
- ◯ 2
- ◯ 3
- ◯ 4
- ◯ 5
- ◯ Food is constantly available

The dog's appetite

Really picky	A bit picky	Normal	A bit greedy	Really greedy
◯	◯	◯	◯	◯

The dog sometimes has a fasting day (by own will or on purpose)

- ◯ Yes
- ◯ No

If yes, how often?

How long has your dog been eating the CURRENT diet?

- ○ His whole life
- ○ All his life, excluding puppy time
- ○ A specific time, how long?

(months/years) [ ]

If you have changed the dog's diet, fill in the information below about the dog's PREVIOUS diet.

☐ I haven't changed my dog's food (if the dog has been eating the same food all his life, you can go to "Material of food and water bowls").

The composition of the dog's PREVIOUS diet (check that all your choices add up to 100%, Thank you!)

	0%	10 %	20 %	30 %	40 %	50 %	60 %	70 %	80 %	90 %	100 %	What (brand/type) ?
Dry food	○	○	○	○	○	○	○	○	○	○	○	[ ]
Raw food/uncooked	○	○	○	○	○	○	○	○	○	○	○	[ ]
Home cooked/table scraps	○	○	○	○	○	○	○	○	○	○	○	[ ]
Dog sausage and canned food	○	○	○	○	○	○	○	○	○	○	○	[ ]

The dog's PREVIOUS diet included

	never	1-6 times a year	monthly	weekly	daily	What (species/brand/type)
Slaughter waste from hunting	○	○	○	◉	○	[ ]
Organs/offal	○	○	○	○	○	[ ]
Fish	○	○	○	◉	○	[ ]
Raw meat bones (e.g., pipe bones, chicken wings,	○	○	○	◉	○	[ ]

	never	1-6 times a year	monthly	weekly	daily	What (species/brand/type)
cartilage bones)						
Boiled rice	○	○	○	○	○	
Vegetables	○	○	○	○	○	
Berries	○	○	○	○	○	
Egg (raw)	○	○	○	○	○	
Egg (cooked)	○	○	○	○	○	
Dairy products	○	○	○	○	○	
Leather bones (white or beige)	○	○	○	○	○	
Treats	○	○	○	○	○	

How long did your dog eat this diet (months/years)? [ ]

What was the reason you changed your diet (from the previous diet to the current diet)?

- [ ] Price
- [ ] Availability
- [ ] Recommended by someone
- [ ] Disease
- [ ] The dog gained weight
- [ ] To get some variability for the dog
- [ ] Flatulence
- [ ] Bad breath
- [ ] Loss of appetite
- [ ] Trying to find a better diet for the digestion
- [ ] Bone problems at a young age
- [ ] For osteoarthritis
- [ ] The food was changed because of another dog
- [ ] The dog always eats a variety of foods

☐ I switch food brands from time to time

Other reason; what? [ ]

If the reason for the food change was illness, what illness?

[ ]

If the reason for the food change was an illness, did the change in feeding help?

Yes	No	I don't know/I don't remember
○	○	○

Material of food and water bowls

☐ Stainless steel

☐ Ceramics

☐ Plastic

Something else, what? [ ]

The dog's drinking water is mainly

☐ Municipal tap water

☐ Water from own well

☐ Spring water

☐ Filtered municipal tap water

☐ Filtered water from own well

Something else, what? [ ]

The dog drinks water

much less than normal	slightly less than normal	normally	a little more than normal	much more than normal
○	○	○	○	○

A dog normally drinks about 0.5 dl of water per kilogram of body weight per day (for example, a dog weighing 10 kg drinks about 0.5 liters (5 dl) of water a day,

a dog weighing 30 kg about 1.5 liters and a dog weighing 50 kg about 2.5 liters). NOTE: if a dog eats raw food, it normally drinks less because the water content of the food is higher than in dry food.

Additional information about the dog's diet

The dog's behavior

How would you describe your dog's character?

- [ ] Perky
- [ ] Cheerful
- [ ] Brave
- [ ] Curious
- [ ] Overactive
- [ ] Calm
- [ ] Blue, seems to be down
- [ ] Nervous
- [ ] Sensitive
- [ ] Aggressive

Something else, what?

How would you describe your dog's attitude?

	Very friendly	Friendly	Indifferent/ retentive	Sensitive	Aggressive	I don't know/I can't say
to the owner?	○	○	○	○	○	○
to other family members?	○	○	○	○	○	○
to strangers?	○	○	○	○	○	○
to other dogs in the family?	○	○	○	○	○	○

	Very friendly	Friendly	Indifferent/ retentive	Sensitive	Aggressive	I don't know/I can't say
strange dogs?	◯	◯	◯	◯	◯	◯

The dog has one of the following behavioral disorders or symptoms

- ☐ Separation anxiety
- ☐ Sound sensitivity
- ☐ Light sensitivity
- ☐ Sensitivity to some types of floor/materials
- ☐ Fearfulness/shyness towards new situations/objects
- ☐ Tail chasing
- ☐ Chasing shadows or lights
- ☐ Hyperactivity
- ☐ Fly snapping
- ☐ Staring seizures

Something else, what? ____________

The dog tends to eat some of the following

- ☐ Grass
- ☐ Sand/soil/mulch from the flower bed
- ☐ Rocks
- ☐ Own poop
- ☐ Other dogs' poop
- ☐ Feces of other animals
- ☐ Metal objects
- ☐ Paint/paint flakes
- ☐ Drywall/cement/mortar

Something else, what? ____________

Additional information about the dog’s behavior

Epilepsy

1. GENERAL INFORMATION ABOUT YOUR DOG'S EPILEPSY

How many hours a day is the dog under your or a family member's supervision (so that possible seizures can be detected)?

- ○ Less than 5 hours/day
- ○ 5-10 hours/day
- ○ 10-15 hours/day
- ○ 15-20 hours/day
- ○ more than 20 hours/day

How old was your dog when it had its first seizure (as accurately as possible)?

How long has it been since your dog's last seizure?

How many seizures has your dog had so far?

How often did your dog's seizures recur in the early stages of the disease?

	Write the number below
Times a day	
Times a week	
Times a month	
Times a year	

Have there been any changes in the seizures since the onset of the illness?

	Reduced significantly	Reduced somewhat	Stayed the same	Increased	Increased significantly
in density?	○	○	○	○	○
in strength?	○	○	○	○	○
in duration?	○	○	○	○	○

How long was the period from the dog's first seizure to the start of epilepsy medication?

- Days
- Weeks
- Months
- Years
- Medication was started immediately after the first seizure
- Medication has not been started at all

What factors do you think increase the occurrence of seizures?

- Stress
- Sexual activity
- Weather
- A certain time of day
- A certain season
- Another factor
- No detectable seizure predisposing factor

If your dog's seizures are affected by a certain time of day or season, could you specify the time in the field below. Also, if you answered above that the seizures are affected by another factor, could you tell us more about it.

If your dog has been castrated/sterilized, did the procedure reduce the occurrence of seizures?

- Yes, the seizures were clearly reduced
- Yes, the seizures decreased somewhat
- Spaying/neutering had no effect on seizure frequency
- No, the seizures increased after the procedure
- I don't know/I can't say
- The dog is not castrated/sterilized

Here you can clarify the answer to the previous question if you wish.

Is the dog completely normal between seizures?

Yes	No	I don't know/I can't say
○	○	○

If you answered 'No', what is the dog like between seizures.

Have the seizures affected the dog's normal behavior?

Yes	No	I don't know/I can't say
○	○	○

If you answered 'Yes', how have the seizures affected the dog's behavior.

Has your dog ever had more than one seizure in 24 hours?

Yes	No	I don't know/I don't remember
○	○	○

If you answered 'Yes', how many times has this happened?

If you answered 'Yes' to the previous question, how many seizures has your dog had within a day?

	Write the number below
Seizures on average	
Seizures minimum	
Seizures maximum	

Does your dog have any relatives with epilepsy?

- Yes
- No
- I don't know/I can't say

If you answered 'Yes', please list your dog's epileptic relatives in the field below (preferably the official name, if you know it).

2. THE PHASE PRIOR TO THE SEIZURE (pre-ictal phase)

This phase refers to the time (hours and days) before the seizure.

What kind of emotional state has the dog been in most often before the seizure?

- At rest
- Sleeping
- Awake in the usual state
- Under strain/exercise
- In the post-exercise state

- [ ] Stressed out
- [ ] Missing the owner
- [ ] Soon after eating
- [ ] After not eating for a long time
- [ ] Sick
- [ ] In a strong emotional state (e.g., angry, in a fight, etc.)
- [ ] At the vet waking up from anesthesia
- [ ] Seizures have mostly started in random situations without being tied to specific emotional states
- [ ] Other emotional state

If another emotional state, what? ______

Can the other dogs in the family predict the onset of the dog's seizure in advance?

Yes	No	I don't know/I can't say	We don't have any other dogs
○	○	○	○

Can you predict the onset of the dog’s seizure in advance?

Yes	No	I don't know/I can't say
○	○	○

If you answered 'No' to the previous question, go directly to point 3. SEIZURE PHASE (ictal phase). If you answered 'Yes', continue below.

What things/symptoms do you notice as signs of an impending seizure?

- [ ] The dog looks sick
- [ ] The dog vomits
- [ ] The dog's saliva production increases and it starts to drool
- [ ] The dog becomes restless
- [ ] The dog wants to be close to the owner
- [ ] The dog becomes aggressive

Please write in the field such things/symptoms that were not found in the options above.

How long before the onset of the seizure are these symptoms noticeable?

- ○ less than 30 min
- ○ 30-60 min
- ○ 1-2 hours
- ○ 2-6 hours
- ○ 6-12 hours
- ○ 12-24 hours
- ○ 1-2 days
- ○ more than 2 days

How often is your prediction of the onset of a seizure correct?

- ○ Never
- ○ 25% of cases
- ○ 50% of cases
- ○ 75% of cases
- ○ Every time

3. SEIZURE PHASE (ictal phase)

This phase refers to the events during the actual seizure and the time immediately before the seizure.

Have you ever seen your dog have a seizure?

--Valitse tästä-- (Yes/No)

Have you ever watched a scene from start to finish?

--Valitse tästä-- (Yes/No)

What does your dog do immediately before a seizure?

☐ Sleeps

☐ Is awake

☐ Is outside

☐ Plays

☐ Exercises with the owner

Something else, what?

Describe in detail the events immediately preceding the seizure.

Have you ever tried to call your dog by name or make contact with him immediately before a seizure?

Yes	No	I don't know/I don't remember
○	○	○

If you answered 'Yes' to the previous question, what was the dog's level of consciousness?

- ○ Completely normal (reacts normally to speech)
- ○ Abnormal but not completely absent (responds in some way to speech or touch)
- ○ Completely absent (does not respond in any way to speech or touch)

How long does the seizure last? (don't take into account the pre-seizure phase or the post-seizure phase)

	minutes
Usually a seizure lasts about	[ ]
The shortest seizure lasted approx	[ ]
The longest seizure lasted approx	[ ]

Rate how typically the options below occur in your dog's seizures (please answer each item).

	Always	Often	Rarely	Never	I don't know/I can't say
Stiffness of limbs and neck	○	○	○	○	○
Falling	○	○	○	○	○
The dog is lying down during the seizure	○	○	○	○	○
Twitching of muscles and limbs	○	○	○	○	○
General tremors	○	○	○	○	○
Turning the head in some direction	○	○	○	○	○
Facial muscle twitching	○	○	○	○	○
Chewing movements	○	○	○	○	○
Urination	○	○	○	○	○
Defecation	○	○	○	○	○
A momentary pause in breathing	○	○	○	○	○
Drooling	○	○	○	○	○
Dilation of the pupils during the seizure	○	○	○	○	○

	Always	Often	Rarely	Never	I don't know/I can't say
Rolling back of the eyes (only white is visible)	○	○	○	○	○
Staring gaze	○	○	○	○	○
Momentary blindness	○	○	○	○	○
Bumping into, for example, furniture	○	○	○	○	○
Going around in circles	○	○	○	○	○
Changing position	○	○	○	○	○
Chasing the tail, etc.	○	○	○	○	○
Snapping invisible flies ("fly snapping")	○	○	○	○	○
Momentary loss of consciousness	○	○	○	○	○
Seeking to be close to people	○	○	○	○	○
Barking	○	○	○	○	○
Fear	○	○	○	○	○
Aggressiveness	○	○	○	○	○

Write down the sequence of events of your dog's important symptoms in numerical order.

Example:
1. Falling
2. Chewing movements
3. Twitching of muscles and limbs
4. Urination

If some of the symptoms occur at the same time, you can use the same number.

Additional information about the seizures:

Are all your dog's seizures the same?

Yes	No	I don't know/I can't say
○	○	○

If you answered 'No', what kind of differences have you noticed?

Have you ever had the impression that one side (left/right) of your dog's body behaves differently than the other side during a seizure? For example, one side twitches more strongly than the other, etc.?

Yes	No	I don't know/I can't say
○	○	○

If you answered 'Yes', please tell us how.

Can you influence the course of a seizure?

Yes	No	I don't know/I can't say
○	○	○

If you answered 'Yes', please tell us how.

4. POST-SEIZURE PHASE (post-ictal phase)

This phase refers to the time from minutes to hours and days after the seizure.

Do you think your dog understands what has happened after the seizure?

Yes	No	I don't know/I can't say
○	○	○

If you answered 'Yes', please tell us how.

Are you afraid of your dog's reactions after a seizure?

Yes	No	I don't know/I can't say
○	○	○

If you answered 'Yes', please tell us why.

Does your dog react to calling/sound immediately after the seizure?

Yes	No	I don't know/I can't say
○	○	○

Have you ever asked your dog to do something/give it a command immediately after a seizure?

Yes	No	I don't know/I don't remember
○	○	○

If you answered 'Yes' to the previous question, what happens?

- ○ The dog obeys normally
- ○ The dog obeys, but behaves abnormally
- ○ The dog does not obey the command

What kinds of things do you notice about your dog in the minutes, hours and days after the seizure?

- ☐ The dog is tired
- ☐ The dog walks around
- ☐ The dog is aggressive
- ☐ The dog drinks
- ☐ The dog eats
- ☐ The dog wants to go out
- ☐ The dog doesn't want to get up
- ☐ The dog feels sick and vomits

If you notice things in your dog other than those listed above, please report them in this field. Also tell the timing of all the things you mentioned above in relation to the seizure.

How long does it take for your dog to return to normal after a seizure?

- The dog behaves normally immediately after the seizure
- Less than 5 minutes
- 5-15 minutes
- 15-30 minutes
- 30-60 minutes
- 1-2 hours
- 2-6 hours
- More than 6 hours

5. VETERINARY EXAMINATIONS AND MEDICATION AND TREATMENT OF EPILEPSY

Has a veterinarian diagnosed your dog with idiopathic epilepsy?

Yes	No	I don't know/I can't say
○	○	○

If you answered 'Yes', when was the disease diagnosed? If you answered 'I don't know/I can't say', you can also provide additional information if you wish.

Were any additional tests done when the dog was diagnosed?

	Yes	No	I don't know/I can't say
Blood test	○	○	○
Electroencephalogram	○	○	○
Cerebrospinal fluid sample	○	○	○
Computed tomography/MRI	○	○	○
Other tests	○	○	○

If you answered 'yes' to the last option, what other tests were done?

Has your dog received another diagnosis for its seizures, such as:

- ☐ Epilepsy
- ☐ Seizures
- ☐ Brain/neurological trauma
- ☐ Other seizure diagnosis

If other seizure diagnosis, what?

Does your dog receive or has it ever received medication to control seizures?

- ○ Yes, and still does
- ○ Yes, but not anymore
- ○ Has never received

If you answered 'Yes' to the previous question, when did your dog start receiving medication?

- ○ As soon as the symptoms started
- ○ When it got seizures more than once a month
- ○ Other time

Find out as precisely as possible how old your dog was when he started eating the medicine, how long has he been eating it and has the medication been continuous?

What is the name of your dog's epilepsy medication?

	Name and strength of the medicine (e.g., Barbivet tablet 30 mg)	Dosage of the medicine (e.g., 2 tablets twice a day)
Medicine 1		
Medicine 2		
Medicine 3		
Medicine 4		
Medicine 5		

Do you administer the medication regularly?

- Yes
- No
- The dog does not receive medication

If you answered 'No', tell us why and in what way irregularly?

Has the concentration of the drug in your dog's blood been measured?

- Yes
- No
- I don't know/I can't say
- The dog does not receive medication

Has the medication reduced the seizures?

- The medication has completely eliminated the seizures
- The medication has reduced the seizures to about half of what they used to be
- The medication has reduced the seizures a little
- The medication has not reduced the number of seizures
- I don't know/I can't say
- The dog does not receive medication

Here, if you wish, you can clarify the answer to the previous question.

Has the medication alleviated the seizures?

- Yes
- No
- I don't know/I can't say
- The dog does not receive medication

If you answered 'Yes', please tell us how.

Do you think the medication affects your dog's performance?

- Yes
- No
- I don't know/I can't say
- The dog does not receive medication

If you answered 'Yes', please tell us how.

Do you medicate your dog during seizures?

- Yes
- No

If you answered yes to the previous question, please tell me which medicine, at what dosage and by what method of administration?

Have you noticed that the medication had side effects?

- I haven't noticed any side effects
- Drowsiness
- Vomiting
- Increased drinking
- Increased urination
- Increased eating
- 'Drunken's rambling'
- Other

If you have noticed that the medication has side effects other than those mentioned above, please report them here.

Have you used any of the following, and did it help with epilepsy symptoms?

	No, we haven't	Yes, but it didn't help	Yes, helped a bit	Yes, helped a lot	If yes, what (e.g., what homeopathy, what herb, what supplement, etc.)
Acupuncture	○	○	○	○	
Gold implants	○	○	○	○	
Osteopathy	○	○	○	○	
Homeopathy	○	○	○	○	
Herbs	○	○	○	○	
Fish oil (omega-3)	○	○	○	○	
Fatty fish (e.g., salmon, trout, herring, roach)	○	○	○	○	
Vegetable oils	○	○	○	○	
Supplements (e.g., vitamins and minerals)	○	○	○	○	

If you have used things other than those listed above, please tell us about them in this field.

Have you noticed that food-related things have affected your dog's epilepsy?

- ☐ A certain food/nutrient has increased the frequency/duration/intensity of the seizures
- ☐ A certain food/nutrient has reduced the frequency/duration/intensity of the attacks

Tell us which food/nutrient, and in more detail how it has affected the disease.

6. OTHER

If you wish, please provide additional information about your dog's epilepsy in the field below.
