# Supplementary material for: Mineral, trace element, and toxic metal concentration in hair from dogs with idiopathic epilepsy compared to healthy controls
Source: J Vet Intern Med. 2023 Apr 6;37(3):1100–10. doi: 10.1111/jvim.16698 (PMC10229330; doi:10.1111/jvim.16698)
Supplement: Supplementary file 3 — Supplementary Material 3. Detailed information about study and control dogs. [file JVIM-37-1100-s003.pdf]

Supplementary Material 3 Detailed information about study and control dogs

Dog nr	Group	Breed	Age (y)	Sex	Weight (kg)	Diet	Age at seizure onset (y)	Time since last seizure	Diagnostic workup for epileptic dogs								ASD
									IE diagnosis (by whom/where)	*The dog shows typical IE symptoms*[i]	*Unremarkable inter-ictal physical and neurological examination*	*Blood tests*	*MRI*	*CSF*	*EEG*	*Other*	
1	treated epileptic	Border Collie[a,b]	3.2	female, spayed	18.0	dry	2.1	1 year	DECVN, HUAH	x	x	c	x	x	x	urinalysis	phenobarbital
2	treated epileptic	Bull Terrier	7.8	female, spayed	16.5	mixed	4.0	4 years	DECVN, HUAH	x	x	c,h	x		x		phenobarbital, potassium bromide
3	treated epileptic	Greater Swiss Mountain Dog[b]	6.9	female, intact	47.0	dry	5.1	1.4 years	unspecified	x	x	c	x		x		phenobarbital
4	treated epileptic	Border Collie[a,b]	5.6	male, neutered	24.0	raw	3.6	1 day	DECVN, EEAH	x	x	d,e,g,h	x	x			phenobarbital, potassium bromide, imepitoin, levetiracetam, topiramate, clorazepate
5	treated epileptic	Cocker Spaniel[a]	3.2	female, intact	12.8	dry	2.3	1 week	DECVN, EEAH	x	x	d,e,h	x	x			phenobarbital, imepitoin
6	treated epileptic	Giant Schnauzer[b]	3.2	male, intact	43.5	dry	2.6	11 days	DECVN, EEAH	x	x	d,e,g,h	x				phenobarbital
7	treated epileptic	Dalmatian[a]	2.3	female, intact	27.0	dry	2.0	4 months	DECVN, EEAH	x	x	d,e,g,h	x				phenobarbital, potassium bromide
8	treated epileptic	Rough Collie	4.9	male, intact	20.0	dry	0.8	5 months	DECVN, EEAH	x	x	d,e,g,h	x				imepitoin
9	treated epileptic	Hungarian Wirehaired Vizsla[a]	5.3	male, intact	32.5	dry	4.0	1.2 years	DECVN, EEAH	x	x	d,e,h	x				phenobarbital
10	treated epileptic	Volpino Italiano	10.7	male, intact	10.4	dry	6.6	2 weeks	DECVN, EEAH	x	x	d,e,h	x				phenobarbital, levetiracetam
11	treated epileptic	Soft-coated Wheaten Terrier	8.5	female, spayed	19.4	dry	0.5	4.5 years	DECVN, EEAH	x	x	d,h	x				phenobarbital, potassium bromide, levetiracetam
12	untreated epileptic	Golden Retriever[a,b]	6.2	female, intact	24.0	dry	4.8	1.4 years	unspecified	x	x	c	x				
13	untreated epileptic	Jack Russel Terrier[a]	3.7	male, intact	8.5	raw	2.8	6 months	unspecified	x	x	c	x				
14	treated epileptic	German Shepherd[a]	6.1	male, intact	41.0	mixed	2.7	3 months	DECVN, HUAH	x	x	c	x				phenobarbital
15	treated epileptic	Red Irish Setter	5.1	female, intact	25.0	mixed	3.8	2 months	DECVN, HUAH	x	x	c	x				imepitoin
16	treated epileptic	Cavalier King Charles Spaniel[a,b]	2.6	male, neutered	12.8	dry	1.8	2 weeks	DECVN, HUAH	x	x	c	x				phenobarbital

17	treated epileptic	Bullmastiff	2.1	male, intact	65.0	raw	1.0	10 months	DECVN, HUAH	x	x	c	x				phenobarbital
18	treated epileptic	Australian Kelpie[b]	6.0	male, neutered	27.0	dry	4.1	4 months	unspecified	x	x	c	x				phenobarbital
19	treated epileptic	Papillon	3.8	female, intact	5.0	mixed	2.7	8 months	unspecified	x	x	c	x				phenobarbital
20	treated epileptic	Petit Basset Griffon Vendéen[a]	3.9	male, intact	16.0	mixed	2.2	3 weeks	unspecified	x	x	c	x				phenobarbital, potassium bromide, levetiracetam
21	treated epileptic	Australian Shepherd[a]	6.2	female, spayed	24.2	mixed	4.1	3 weeks	DECVN, HUAH	x	x	c	x				phenobarbital, potassium bromide
22	treated epileptic	White Swiss Shepherd Dog	5.0	female, intact	30.0	raw	3.0	0.5 year	unspecified	x	x	c	x				phenobarbital
23	treated epileptic	Australian Shepherd[a]	3.6	female, intact	24.0	mixed	2.2	1 month	DECVN, EEAH	x	x	d,e,g,h					imepitoin
24	treated epileptic	Saint Bernard	4.5	female, intact	65.0	dry	4.1	2 weeks	DECVN, EEAH	x	x	d,e,g,h					phenobarbital, potassium bromide
25	treated epileptic	Flat-coated Retriever	5.2	male, neutered	32.5	dry	2.1	1.5 years	DECVN, EEAH	x	x	d,e,g,h				urinalysis	phenobarbital
26	treated epileptic	Border Collie[a]	4.2	male, neutered	18.0	dry	3.0	1 year	DECVN, EEAH	x	x	d,e,h					phenobarbital
27	treated epileptic	Australian Labradoodle[b]	3.7	male, neutered	27.1	dry	2.0	4 months	DECVN, EEAH	x	x	d,e,h					phenobarbital
28	treated epileptic	Mixed breed	5.9	male, neutered	17.7	mixed	0.6	1.5 weeks	DECVN, EEAH	x	x	d,e,h					phenobarbital, potassium bromide, levetiracetam
29	untreated epileptic	Golden Retriever[a]	3.6	male, neutered	33.5	dry	3.3	2 weeks	DECVN, EEAH	x	x	d,e,h					
30	treated epileptic	Border Terrier[a]	8.2	female, intact	10.8	dry	3.0	2 months	DECVN, EEAH	x	x	d,e,h					potassium bromide
31	treated epileptic	Australian Terrier	1.6	female, spayed	8.5	mixed	0.6	1 year	DECVN, HUAH	x	x	c,f					imepitoin
32	treated epileptic	Mixed breed[b]	4.5	male, intact	30.0	dry	1.8	2 hours	DECVN, HUAH	x	x	c,f				urinalysis	phenobarbital, potassium bromide, levetiracetam
33	treated epileptic	Cavalier King Charles Spaniel[a]	2.4	female, intact	8.0	dry	1.7	1 month	unspecified	x	x	c,e					phenobarbital
34	untreated epileptic	Labrador Retriever[a]	4.0	male, intact	36.0	dry	2.3	6 days	unspecified	x	x	c					
35	untreated epileptic	Black Russian Terrier	2.4	male, intact	57.0	dry	2.3	2 hours	unspecified	x	x	c					
36	untreated epileptic	Finnish Spitz[a,b]	8.1	female, intact	15.0	dry	3.2	7 months	unspecified	x	x	c					
37	untreated epileptic	Siberian Husky[b]	4.9	male, neutered	23.0	mixed	4.8	1 day	unspecified	x	x	c					
38	untreated epileptic	Italian Greyhound	3.4	male, intact	7.0	dry	1.5	8 days	unspecified	x	x	c					
39	untreated epileptic	Australian Shepherd[a,b]	8.3	female, intact	20.0	mixed	4.2	3.5 years	unspecified	x	x	c					
40	untreated epileptic	Finnish Lapphund	6.7	male, intact	19.0	raw	3.0	3 months	unspecified	x	x	c					

41	treated epileptic	Lagotto Romagnolo[a]	4.1	male, neutered	20.7	mixed	0.8	4 months	DECVN, HUAH	x	x	c					phenobarbital
42	treated epileptic	Bichon Frisé	10.2	male, neutered	6.0	mixed	3.0	1 year	unspecified	x	x	c					phenobarbital
43	treated epileptic	Belgian Shepherd Tervueren[a,b]	7.2	male, neutered	26.0	dry	1.0	1 month	unspecified	x	x	c					phenobarbital
44	treated epileptic	Miniature Pinscher[b]	10.3	male, neutered	5.0	raw	1.3	1.2 years	unspecified	x	x	c					imepitoin
45	treated epileptic	Belgian Shepherd Groenendael[a,b]	2.5	male, intact	23.0	dry	1.6	7 months	unspecified	x	x	c					phenobarbital
46	treated epileptic	Whippet[b]	6.7	female, spayed	15.0	mixed	3.0	5.5 months	unspecified	x	x	c					phenobarbital, potassium bromide
47	treated epileptic	Pyrenean Mastiff[b]	4.0	male, intact	68.0	dry	2.0	4 months	unspecified	x	x	c					phenobarbital
48	treated epileptic	Beagle[a,b]	7.0	male, intact	20.0	dry	5.5	1 hour	unspecified	x	x	c					phenobarbital
49	treated epileptic	Kromfohrländer[b]	5.8	male, neutered	14.0	mixed	1.5	3 days	unspecified	x	x	c					phenobarbital
50	treated epileptic	Mixed breed	6.8	male, neutered	25.0	mixed	0.5	1 year	unspecified	x	x	c					phenobarbital, potassium bromide
51	treated epileptic	German Shorthaired Pointer[b]	2.0	male, neutered	25.0	mixed	1.1	3.5 months	unspecified	x	x	c					phenobarbital
52	treated epileptic	Staffordshire Bull Terrier	2.8	male, neutered	19.0	mixed	1.5	1 year	unspecified	x	x	c					phenobarbital
53	treated epileptic	Dalmatian[a]	4.3	male, neutered	34.0	dry	3.0	1 month	unspecified	x	x	c					phenobarbital
54	treated epileptic	Basenji[b]	3.3	female, intact	9.2	mixed	3.0	1 week	DECVN, HUAH	x	x	c					phenobarbital
55	treated epileptic	Kooikerhondje[b]	7.9	male, intact	10.0	dry	1.6	1.4 years	unspecified	x	x	c				urinalysis	phenobarbital
56	treated epileptic	Malinois	6.9	male, neutered	24.0	dry	5.4	1.5 weeks	unspecified	x	x	c				urinalysis	phenobarbital
57	treated epileptic	Jack Russel Terrier[a]	2.2	male, intact	9.6	dry	1.8	4 months	unspecified	x	x	c					phenobarbital
58	treated epileptic	Long-haired Chihuahua	11.6	female, spayed	1.0	raw	5.0	2 weeks	unspecified	x	x	c					imepitoin
59	treated epileptic	Australian Kelpie[b]	4.8	male, intact	28.0	mixed	4.1	1 month	unspecified	x	x	c					phenobarbital
60	treated epileptic	Border Collie[a,b]	8.7	female, spayed	17.8	mixed	5.0	1 year	unspecified	x	x	c					phenobarbital
61	treated epileptic	Hungarian Smooth-Haired Vizsla[a]	2.0	male, neutered	25.0	mixed	0.9	1.5 months	unspecified	x	x	c					phenobarbital
62	treated epileptic	Russian Toy Terrier	6.8	female, intact	2.0	mixed	3.0	3 months	general practitioner, HUAH	x	x	c					phenobarbital, potassium bromide
63	treated epileptic	Belgian Shepherd Groenendael[a,b]	11.2	female, spayed	22.0	raw	1.0	2.5 months	unspecified	x	x	c				urinalysis	phenobarbital

64	healthy	Labrador Retriever	7.5	male, intact	39.0	dry											
65	healthy	Alaskan Malamute	4.3	male, intact	38.0	mixed											
66	healthy	Australian Kelpie	3.5	male, intact	16.0	mixed											
67	healthy	Australian Kelpie	9.2	male, intact	22.0	dry											
68	healthy	Finnish Spitz	11.4	female, intact	16.0	dry											
69	healthy	Finnish Spitz	8.9	male, intact	16.0	dry											
70	healthy	Finnish Spitz	6.5	female, intact	12.0	dry											
71	healthy	Finnish Spitz	6.8	male, intact	12.0	dry											
72	healthy	Petit Basset Griffon Vendéen	7.0	male, intact	17.0	mixed											
73	healthy	Malinois	5.6	male, intact	28.0	dry											
74	healthy	Jack Russel Terrier	7.5	female, spayed	5.2	mixed											
75	healthy	Australian Shepherd	6.1	male, intact	25.0	dry											
76	healthy	Mixed breed	6.0	male, intact	25.0	dry											
77	healthy	Mixed breed	9.6	male, neutered	22.0	dry											
78	healthy	Norwegian Elkhound	9.3	female, spayed	34.9	dry											
79	healthy	Mixed breed	4.1	male, neutered	21.0	dry											
80	healthy	Mixed breed	6.0	male, neutered	29.0	dry											
81	healthy	German Shepherd	5.2	female, intact	29.0	mixed											
82	healthy	Czech Mountain Dog	4.1	female, spayed	20.0	dry											
83	healthy	Mixed breed	3.7	male, neutered	10.8	mixed											
84	healthy	Australian Kelpie	7.3	male, neutered	23.0	dry											
85	healthy	Pumi	12.1	female, intact	11.5	mixed											
86	healthy	Cavalier King Charles Spaniel	8.5	female, intact	10.0	dry											
87	healthy	Finnish Lapphund	5.4	female, intact	18.0	mixed											
88	healthy	Mixed breed	3.2	female, intact	25.0	mixed											

89	healthy	Slovak Cuvac	10.0	female, intact	34.5	raw											
90	healthy	French Bulldog	3.8	male, intact	15.5	raw											
91	healthy	Border Collie	5.8	female, intact	16.0	raw											
92	healthy	Border Collie	4.3	female, intact	17.0	raw											
93	healthy	Mixed breed	5.2	female, spayed	20.0	raw											
94	healthy	Mixed breed	3.5	male, neutered	33.8	raw											
95	healthy	Bernese Mountain Dog	3.0	female, intact	37.0	dry											
96	healthy	Cocker Spaniel	3.7	female, intact	18.0	mixed											
97	healthy	White Swiss Shepherd Dog	3.3	female, intact	27.4	raw											
98	healthy	White Swiss Shepherd Dog	8.4	female, intact	33.4	raw											
99	healthy	Gordon Setter	10.6	male, intact	35.3	raw											
100	healthy	Gordon Setter	8.0	male, intact	38.9	raw											
101	healthy	Nova Scotia Duck Tolling Retriever	10.8	female, intact	14.6	raw											
102	healthy	Nova Scotia Duck Tolling Retriever	3.2	male, intact	20.6	raw											
103	healthy	Mixed breed	6.8	female, spayed	14.3	dry											
104	healthy	Bullmastiff	3.7	male, intact	50.1	dry											
105	healthy	Kromfohrländer	6.1	male, intact	10.0	dry											

ASD, antiseizure drug; CSF, cerebrospinal fluid analysis; DECVN, Diplomate of European College of Veterinary Neurology; EEAH, Evidensia Espoo Animal Hospital; EEG, electroencephalogram; HUAH, Helsinki University Animal Hospital; IE, idiopathic epilepsy; MRI, magnetic resonance imaging; y, years.

[a]epilepsy prone breed according to literature (ref. 4,25)

[b]family history of idiopathic epilepsy reported by owner in questionnaire

c: complete blood cell count (leucocytes, erythrocytes, hemoglobin, hematocrit, mean cell volume, mean cell hemoglobin, mean corpuscular hemoglobin concentration, and thrombocytes) and basic serum biochemistry (alkaline phosphatase, alanine aminotransferase, albumin, total bilirubin, phosphate, glucose, potassium, sodium, calcium, cholesterol, creatinine, protein, and urea)

d: same as c, but additionally reticulocytes, symmetric dimethylarginine, chloride, gamma glutamyl transferase, aspartate aminotransferase, glutamate dehydrogenase, globulin, a-amylase, lipase, fructosamine, muscle creatine kinase, magnesium, triglycerides, c-reactive protein, basophils, eosinophils, segmented neutrophils, lymphocytes, and monocytes

e: fasting and post-prandial bile acids

f: ammonia

g: vector-borne pathogens (Anaplasma, Lyme disease, Ehrlichia, heartworm)

h: thyroid profile (thyroxine, free thyroxine, thyrotropin, and thyroxine/thyrotropin) or total thyroxinei: The dog shows two or more of the following symptoms that are typical for focal or generalized seizures with tonic-clonic movements and loss of or impaired consciousness: stiffness of limbs and neck, falling, lying down, twitching of muscles and limbs, turning the head in some direction, facial muscle twitching, chewing movements, urination, defecation, drooling, dilation of the pupils, rolling back of the eyes.
